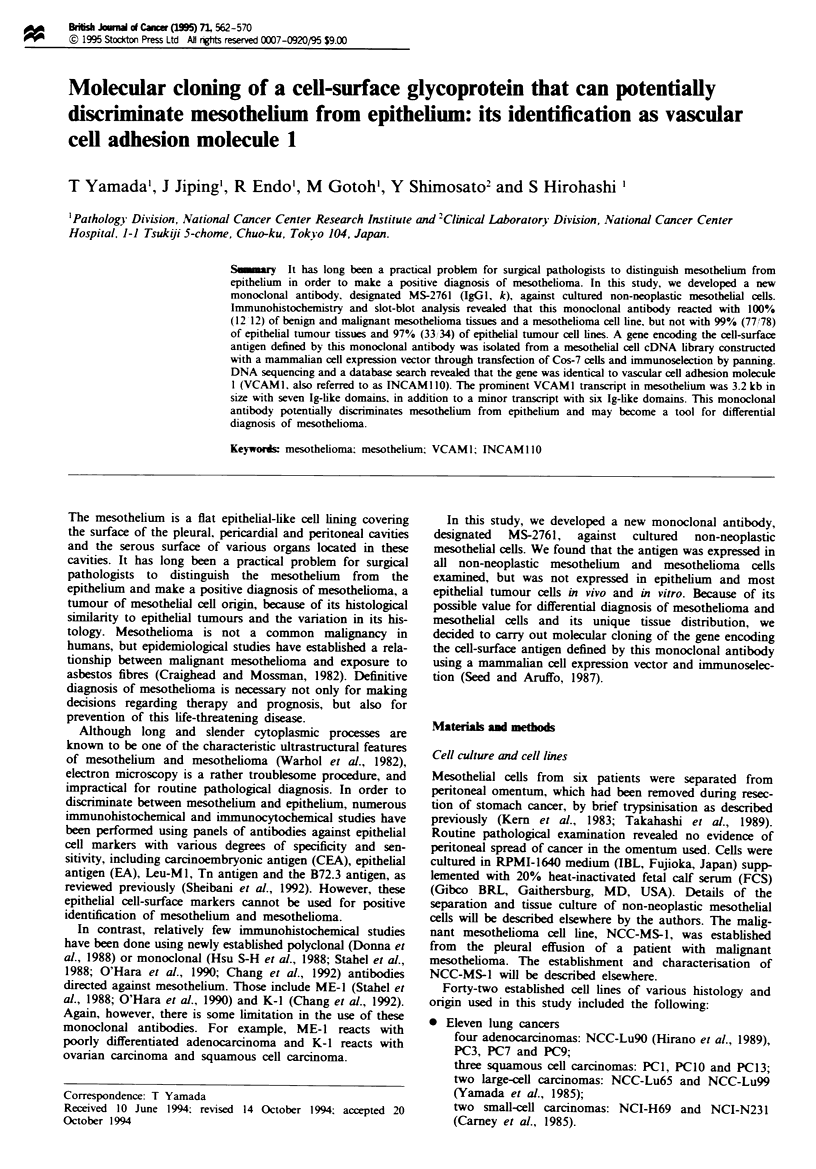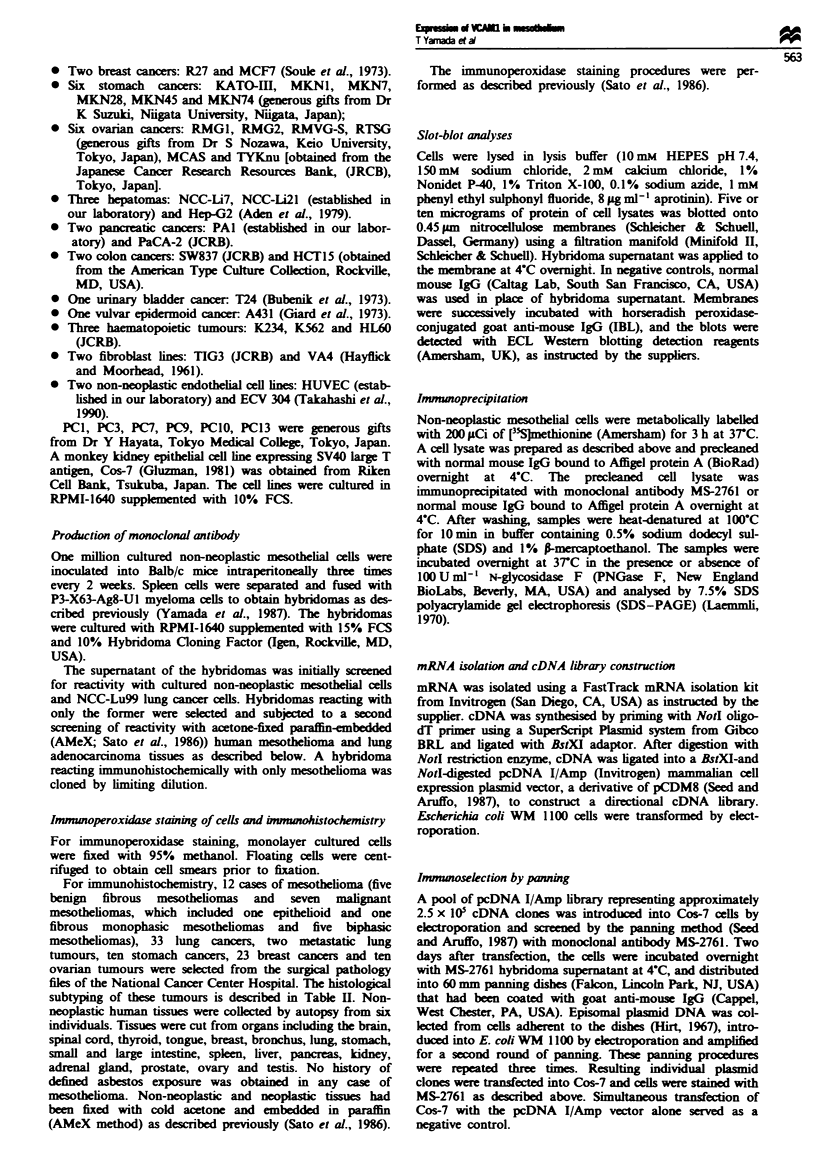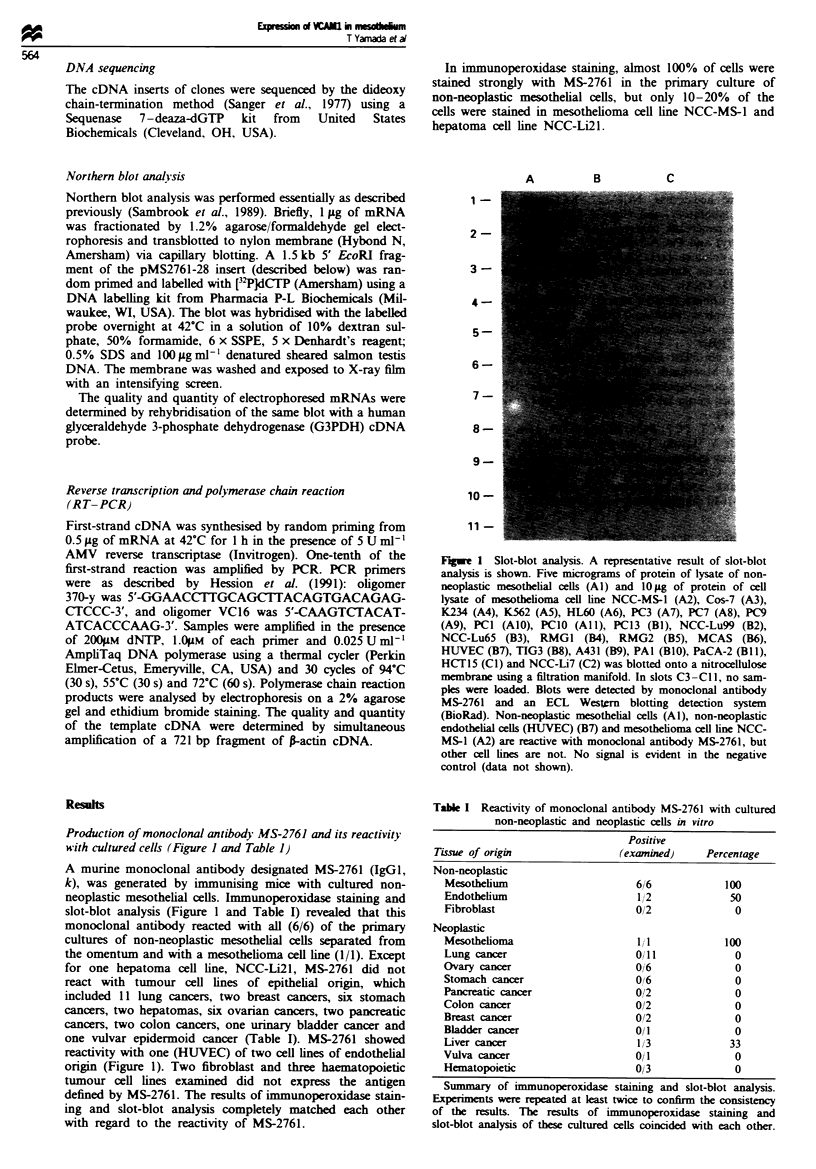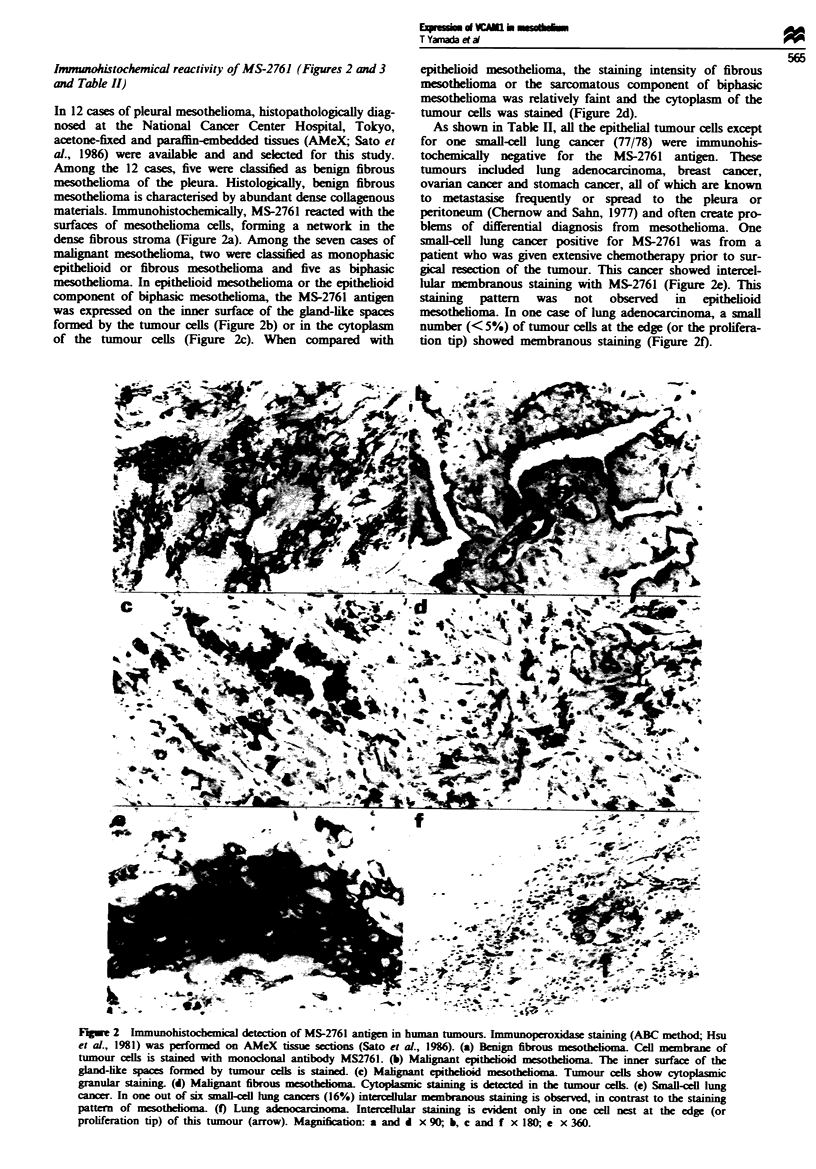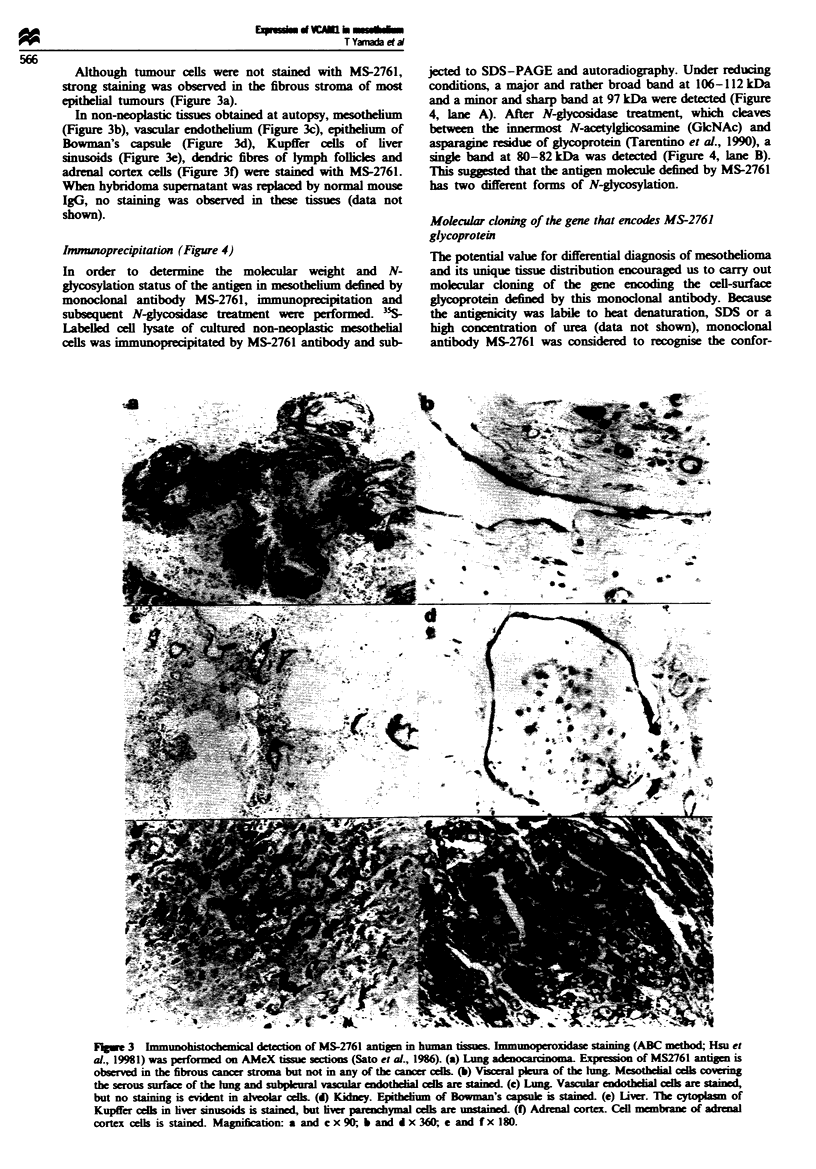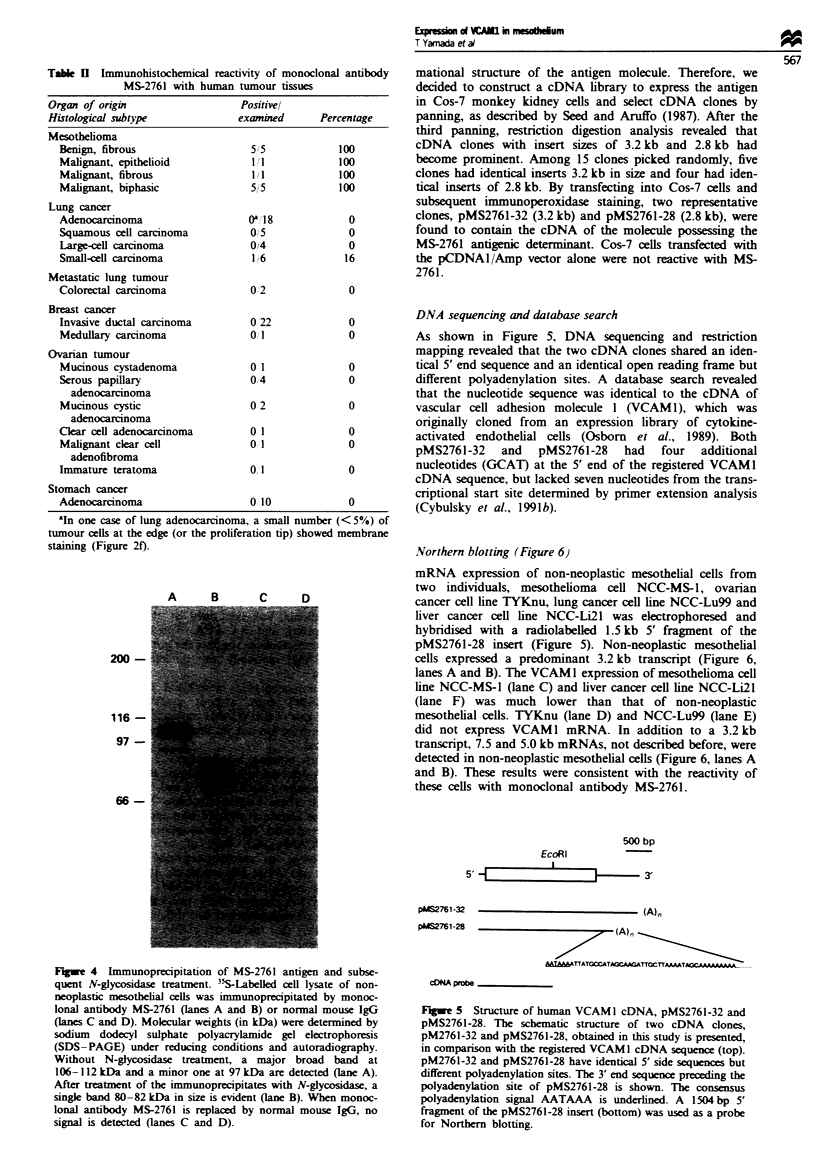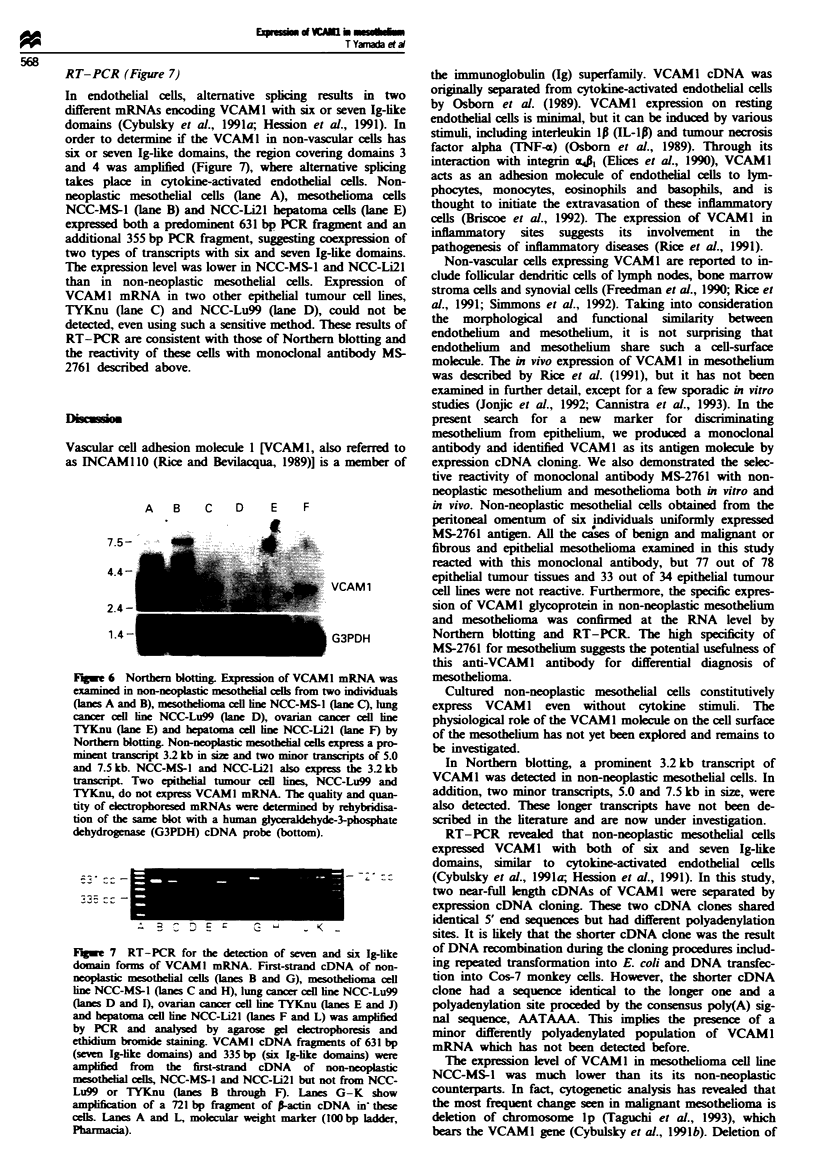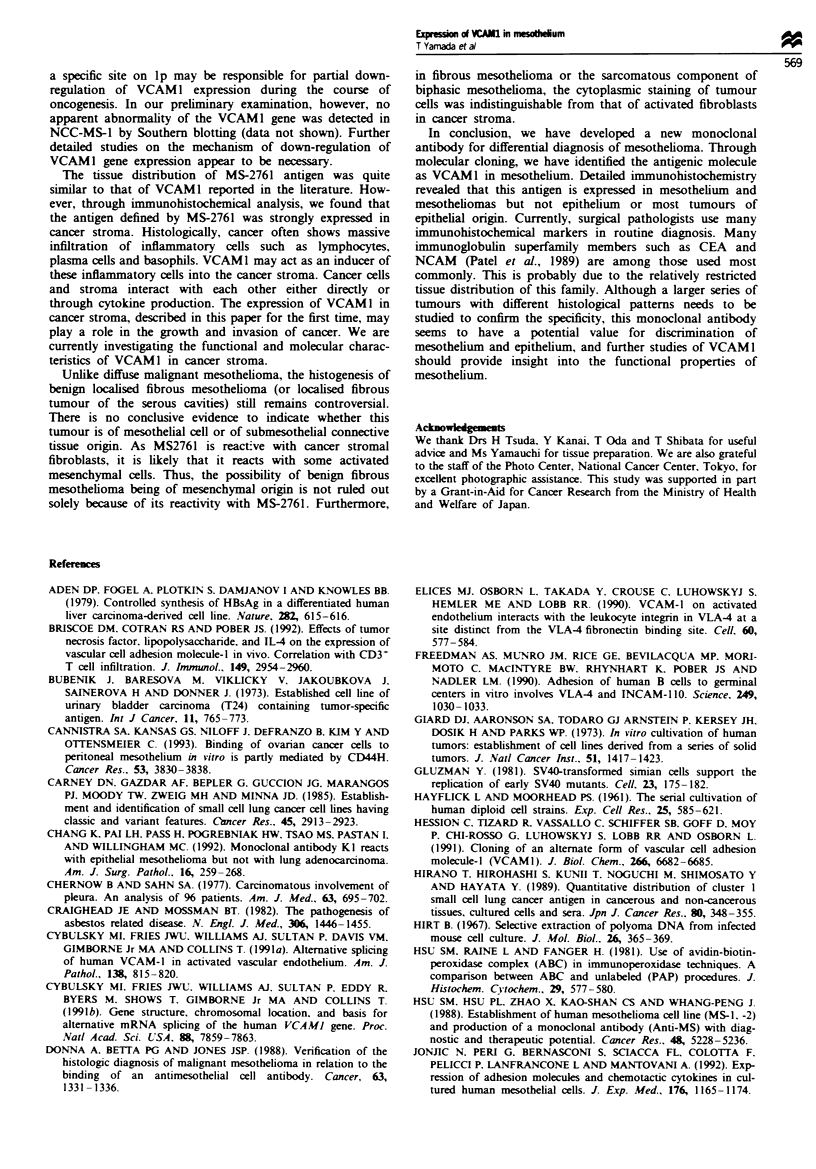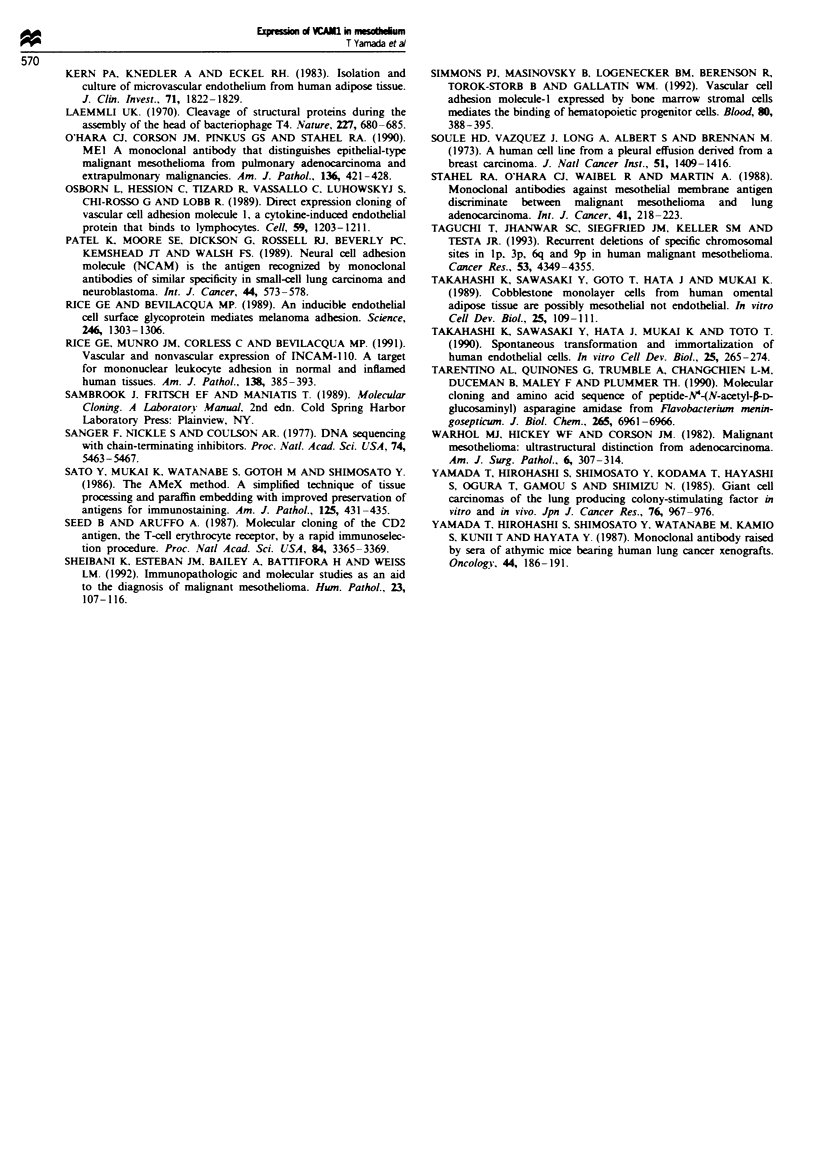# Molecular cloning of a cell-surface glycoprotein that can potentially discriminate mesothelium from epithelium: its identification as vascular cell adhesion molecule 1.

**DOI:** 10.1038/bjc.1995.110

**Published:** 1995-03

**Authors:** T. Yamada, J. Jiping, R. Endo, M. Gotoh, Y. Shimosato, S. Hirohashi

**Affiliations:** Pathology Division, National Cancer Center Hospital, Tokyo, Japan.

## Abstract

**Images:**


British Journal of Cancer (1995) 71, 562-570

© 1995 Stockton Press Ltd All rights reserved 0007-0920/95 $9.00

### Molecular cloning of a cell-surface glycoprotein that can potentially discriminate mesothelium from epithelium: its identification as vascular cell adhesion molecule 1

T Yamada[1], J Jiping[1], R Endo[1], M Gotoh[1], Y Shimosato[2] and S Hirohashi [1]

[1]*Pathology Division, National Cancer Center Research Institute and* [2]*Clinical Laboratory Division, National Cancer Center Hospital, 1-1 Tsukiji 5-chome, Chuo-ku, Tokyo 104, Japan.*

**Summary** It has long been a practical problem for surgical pathologists to distinguish mesothelium from epithelium in order to make a positive diagnosis of mesothelioma. In this study, we developed a new monoclonal antibody, designated MS-2761 (IgG1, *k*), against cultured non-neoplastic mesothelial cells. Immunohistochemistry and slot-blot analysis revealed that this monoclonal antibody reacted with 100% (12/12) of benign and malignant mesothelioma tissues and a mesothelioma cell line, but not with 99% (77/78) of epithelial tumour tissues and 97% (33/34) of epithelial tumour cell lines. A gene encoding the cell-surface antigen defined by this monoclonal antibody was isolated from a mesothelial cell cDNA library constructed with a mammalian cell expression vector through transfection of Cos-7 cells and immunoselection by panning. DNA sequencing and a database search revealed that the gene was identical to vascular cell adhesion molecule 1 (VCAM1, also referred to as INCAM110). The prominent VCAM1 transcript in mesothelium was 3.2 kb in size with seven Ig-like domains, in addition to a minor transcript with six Ig-like domains. This monoclonal antibody potentially discriminates mesothelium from epithelium and may become a tool for differential diagnosis of mesothelioma.

**Keywords:** mesothelioma; mesothelium; VCAM1; INCAM110

The mesothelium is a flat epithelial-like cell lining covering the surface of the pleural, pericardial and peritoneal cavities and the serous surface of various organs located in these cavities. It has long been a practical problem for surgical pathologists to distinguish the mesothelium from the epithelium and make a positive diagnosis of mesothelioma, a tumour of mesothelial cell origin, because of its histological similarity to epithelial tumours and the variation in its histology. Mesothelioma is not a common malignancy in humans, but epidemiological studies have established a relationship between malignant mesothelioma and exposure to asbestos fibres (Craighead and Mossman, 1982). Definitive diagnosis of mesothelioma is necessary not only for making decisions regarding therapy and prognosis, but also for prevention of this life-threatening disease.

Although long and slender cytoplasmic processes are known to be one of the characteristic ultrastructural features of mesothelium and mesothelioma (Warhol *et al.*, 1982), electron microscopy is a rather troublesome procedure, and impractical for routine pathological diagnosis. In order to discriminate between mesothelium and epithelium, numerous immunohistochemical and immunocytochemical studies have been performed using panels of antibodies against epithelial cell markers with various degrees of specificity and sensitivity, including carcinoembryonic antigen (CEA), epithelial antigen (EA), Leu-M1, Tn antigen and the B72.3 antigen, as reviewed previously (Sheibani *et al.*, 1992). However, these epithelial cell-surface markers cannot be used for positive identification of mesothelium and mesothelioma.

In contrast, relatively few immunohistochemical studies have been done using newly established polyclonal (Donna *et al.*, 1988) or monoclonal (Hsu S-H *et al.*, 1988; Stahel *et al.*, 1988; O'Hara *et al.*, 1990; Chang *et al.*, 1992) antibodies directed against mesothelium. Those include ME-1 (Stahel *et al.*, 1988; O'Hara *et al.*, 1990) and K-1 (Chang *et al.*, 1992). Again, however, there is some limitation in the use of these monoclonal antibodies. For example, ME-1 reacts with poorly differentiated adenocarcinoma and K-1 reacts with ovarian carcinoma and squamous cell carcinoma.

Correspondence: T Yamada
Received 10 June 1994; revised 14 October 1994; accepted 20 October 1994

In this study, we developed a new monoclonal antibody, designated MS-2761, against cultured non-neoplastic mesothelial cells. We found that the antigen was expressed in all non-neoplastic mesothelium and mesothelioma cells examined, but was not expressed in epithelium and most epithelial tumour cells *in vivo* and *in vitro*. Because of its possible value for differential diagnosis of mesothelioma and mesothelial cells and its unique tissue distribution, we decided to carry out molecular cloning of the gene encoding the cell-surface antigen defined by this monoclonal antibody using a mammalian cell expression vector and immunoselection (Seed and Aruffo, 1987).

### Materials and methods

### *Cell culture and cell lines*

Mesothelial cells from six patients were separated from peritoneal omentum, which had been removed during resection of stomach cancer, by brief trypsinisation as described previously (Kern *et al.*, 1983; Takahashi *et al.*, 1989). Routine pathological examination revealed no evidence of peritoneal spread of cancer in the omentum used. Cells were cultured in RPMI-1640 medium (IBL, Fujioka, Japan) supplemented with 20% heat-inactivated fetal calf serum (FCS) (Gibco BRL, Gaithersburg, MD, USA). Details of the separation and tissue culture of non-neoplastic mesothelial cells will be described elsewhere by the authors. The malignant mesothelioma cell line, NCC-MS-1, was established from the pleural effusion of a patient with malignant mesothelioma. The establishment and characterisation of NCC-MS-1 will be described elsewhere.

Forty-two established cell lines of various histology and origin used in this study included the following:

- Eleven lung cancers
  four adenocarcinomas: NCC-Lu90 (Hirano *et al.*, 1989), PC3, PC7 and PC9;
  three squamous cell carcinomas: PC1, PC10 and PC13;
  two large-cell carcinomas: NCC-Lu65 and NCC-Lu99 (Yamada *et al.*, 1985);
  two small-cell carcinomas: NCI-H69 and NCI-N231 (Carney *et al.*, 1985).

- Two breast cancers: R27 and MCF7 (Soule *et al.*, 1973).
- Six stomach cancers: KATO-III, MKN1, MKN7, MKN28, MKN45 and MKN74 (generous gifts from Dr K Suzuki, Niigata University, Niigata, Japan);
- Six ovarian cancers: RMG1, RMG2, RMVG-S, RTSG (generous gifts from Dr S Nozawa, Keio University, Tokyo, Japan), MCAS and TYKnu [obtained from the Japanese Cancer Research Resources Bank, (JRCB), Tokyo, Japan].
- Three hepatomas: NCC-Li7, NCC-Li21 (established in our laboratory) and Hep-G2 (Aden *et al.*, 1979).
- Two pancreatic cancers: PA1 (established in our laboratory) and PaCA-2 (JCRB).
- Two colon cancers: SW837 (JCRB) and HCT15 (obtained from the American Type Culture Collection, Rockville, MD, USA).
- One urinary bladder cancer: T24 (Bubenik *et al.*, 1973).
- One vulvar epidermoid cancer: A431 (Giard *et al.*, 1973).
- Three haematopoietic tumours: K234, K562 and HL60 (JCRB).
- Two fibroblast lines: TIG3 (JCRB) and VA4 (Hayflick and Moorhead, 1961).
- Two non-neoplastic endothelial cell lines: HUVEC (established in our laboratory) and ECV 304 (Takahashi *et al.*, 1990).

PC1, PC3, PC7, PC9, PC10, PC13 were generous gifts from Dr Y Hayata, Tokyo Medical College, Tokyo, Japan. A monkey kidney epithelial cell line expressing SV40 large T antigen, Cos-7 (Gluzman, 1981) was obtained from Riken Cell Bank, Tsukuba, Japan. The cell lines were cultured in RPMI-1640 supplemented with 10% FCS.

*Production of monoclonal antibody*

One million cultured non-neoplastic mesothelial cells were inoculated into Balb/c mice intraperitoneally three times every 2 weeks. Spleen cells were separated and fused with P3-X63-Ag8-U1 myeloma cells to obtain hybridomas as described previously (Yamada *et al.*, 1987). The hybridomas were cultured with RPMI-1640 supplemented with 15% FCS and 10% Hybridoma Cloning Factor (Igen, Rockville, MD, USA).

The supernatant of the hybridomas was initially screened for reactivity with cultured non-neoplastic mesothelial cells and NCC-Lu99 lung cancer cells. Hybridomas reacting with only the former were selected and subjected to a second screening of reactivity with acetone-fixed paraffin-embedded (AMeX; Sato *et al.*, 1986)) human mesothelioma and lung adenocarcinoma tissues as described below. A hybridoma reacting immunohistochemically with only mesothelioma was cloned by limiting dilution.

*Immunoperoxidase staining of cells and immunohistochemistry*

For immunoperoxidase staining, monolayer cultured cells were fixed with 95% methanol. Floating cells were centrifuged to obtain cell smears prior to fixation.

For immunohistochemistry, 12 cases of mesothelioma (five benign fibrous mesotheliomas and seven malignant mesotheliomas, which included one epithelioid and one fibrous monophasic mesotheliomas and five biphasic mesotheliomas), 33 lung cancers, two metastatic lung tumours, ten stomach cancers, 23 breast cancers and ten ovarian tumours were selected from the surgical pathology files of the National Cancer Center Hospital. The histological subtyping of these tumours is described in Table II. Non-neoplastic human tissues were collected by autopsy from six individuals. Tissues were cut from organs including the brain, spinal cord, thyroid, tongue, breast, bronchus, lung, stomach, small and large intestine, spleen, liver, pancreas, kidney, adrenal gland, prostate, ovary and testis. No history of defined asbestos exposure was obtained in any case of mesothelioma. Non-neoplastic and neoplastic tissues had been fixed with cold acetone and embedded in paraffin (AMeX method) as described previously (Sato *et al.*, 1986).

The immunoperoxidase staining procedures were performed as described previously (Sato *et al.*, 1986).

*Slot-blot analyses*

Cells were lysed in lysis buffer (10 mM HEPES pH 7.4, 150 mM sodium chloride, 2 mM calcium chloride, 1% Nonidet P-40, 1% Triton X-100, 0.1% sodium azide, 1 mM phenyl ethyl sulphonyl fluoride, 8 $\mu g\ ml^{-1}$ aprotinin). Five or ten micrograms of protein of cell lysates was blotted onto 0.45 μm nitrocellulose membranes (Schleicher & Schuell, Dassel, Germany) using a filtration manifold (Minifold II, Schleicher & Schuell). Hybridoma supernatant was applied to the membrane at 4°C overnight. In negative controls, normal mouse IgG (Caltag Lab, South San Francisco, CA, USA) was used in place of hybridoma supernatant. Membranes were successively incubated with horseradish peroxidase-conjugated goat anti-mouse IgG (IBL), and the blots were detected with ECL Western blotting detection reagents (Amersham, UK), as instructed by the suppliers.

*Immunoprecipitation*

Non-neoplastic mesothelial cells were metabolically labelled with 200 μCi of [$^{35}$S]methionine (Amersham) for 3 h at 37°C. A cell lysate was prepared as described above and precleaned with normal mouse IgG bound to Affigel protein A (BioRad) overnight at 4°C. The precleaned cell lysate was immunoprecipitated with monoclonal antibody MS-2761 or normal mouse IgG bound to Affigel protein A overnight at 4°C. After washing, samples were heat-denatured at 100°C for 10 min in buffer containing 0.5% sodium dodecyl sulphate (SDS) and 1% β-mercaptoethanol. The samples were incubated overnight at 37°C in the presence or absence of 100 $U\ ml^{-1}$ N-glycosidase F (PNGase F, New England BioLabs, Beverly, MA, USA) and analysed by 7.5% SDS polyacrylamide gel electrophoresis (SDS-PAGE) (Laemmli, 1970).

*mRNA isolation and cDNA library construction*

mRNA was isolated using a FastTrack mRNA isolation kit from Invitrogen (San Diego, CA, USA) as instructed by the supplier. cDNA was synthesised by priming with *Not*I oligo-dT primer using a SuperScript Plasmid system from Gibco BRL and ligated with *Bst*XI adaptor. After digestion with *Not*I restriction enzyme, cDNA was ligated into a *Bst*XI-and *Not*I-digested pcDNA I/Amp (Invitrogen) mammalian cell expression plasmid vector, a derivative of pCDM8 (Seed and Aruffo, 1987), to construct a directional cDNA library. *Escherichia coli* WM 1100 cells were transformed by electroporation.

*Immunoselection by panning*

A pool of pcDNA I/Amp library representing approximately $2.5 \times 10^5$ cDNA clones was introduced into Cos-7 cells by electroporation and screened by the panning method (Seed and Aruffo, 1987) with monoclonal antibody MS-2761. Two days after transfection, the cells were incubated overnight with MS-2761 hybridoma supernatant at 4°C, and distributed into 60 mm panning dishes (Falcon, Lincoln Park, NJ, USA) that had been coated with goat anti-mouse IgG (Cappel, West Chester, PA, USA). Episomal plasmid DNA was collected from cells adherent to the dishes (Hirt, 1967), introduced into *E. coli* WM 1100 by electroporation and amplified for a second round of panning. These panning procedures were repeated three times. Resulting individual plasmid clones were transfected into Cos-7 and cells were stained with MS-2761 as described above. Simultaneous transfection of Cos-7 with the pcDNA I/Amp vector alone served as a negative control.

*DNA sequencing*

The cDNA inserts of clones were sequenced by the dideoxy chain-termination method (Sanger *et al.*, 1977) using a Sequenase 7-deaza-dGTP kit from United States Biochemicals (Cleveland, OH, USA).

*Northern blot analysis*

Northern blot analysis was performed essentially as described previously (Sambrook *et al.*, 1989). Briefly, 1 μg of mRNA was fractionated by 1.2% agarose/formaldehyde gel electrophoresis and transblotted to nylon membrane (Hybond N, Amersham) via capillary blotting. A 1.5 kb 5′ *Eco*RI fragment of the pMS2761-28 insert (described below) was random primed and labelled with [$^{32}$P]dCTP (Amersham) using a DNA labelling kit from Pharmacia P-L Biochemicals (Milwaukee, WI, USA). The blot was hybridised with the labelled probe overnight at 42°C in a solution of 10% dextran sulphate, 50% formamide, 6 × SSPE, 5 × Denhardt's reagent; 0.5% SDS and 100 μg ml$^{-1}$ denatured sheared salmon testis DNA. The membrane was washed and exposed to X-ray film with an intensifying screen.

The quality and quantity of electrophoresed mRNAs were determined by rehybridisation of the same blot with a human glyceraldehyde 3-phosphate dehydrogenase (G3PDH) cDNA probe.

*Reverse transcription and polymerase chain reaction (RT–PCR)*

First-strand cDNA was synthesised by random priming from 0.5 μg of mRNA at 42°C for 1 h in the presence of 5 U ml$^{-1}$ AMV reverse transcriptase (Invitrogen). One-tenth of the first-strand reaction was amplified by PCR. PCR primers were as described by Hession *et al.* (1991): oligomer 370-y was 5′-GGAACCTTGCAGCTTACAGTGACAGAG-CTCCC-3′, and oligomer VC16 was 5′-CAAGTCTACAT-ATCACCCAAG-3′. Samples were amplified in the presence of 200μM dNTP, 1.0μM of each primer and 0.025 U ml$^{-1}$ AmpliTaq DNA polymerase using a thermal cycler (Perkin Elmer-Cetus, Emeryville, CA, USA) and 30 cycles of 94°C (30 s), 55°C (30 s) and 72°C (60 s). Polymerase chain reaction products were analysed by electrophoresis on a 2% agarose gel and ethidium bromide staining. The quality and quantity of the template cDNA were determined by simultaneous amplification of a 721 bp fragment of β-actin cDNA.

### Results

*Production of monoclonal antibody MS-2761 and its reactivity with cultured cells (Figure 1 and Table 1)*

A murine monoclonal antibody designated MS-2761 (IgG1, *k*), was generated by immunising mice with cultured non-neoplastic mesothelial cells. Immunoperoxidase staining and slot-blot analysis (Figure 1 and Table I) revealed that this monoclonal antibody reacted with all (6/6) of the primary cultures of non-neoplastic mesothelial cells separated from the omentum and with a mesothelioma cell line (1/1). Except for one hepatoma cell line, NCC-Li21, MS-2761 did not react with tumour cell lines of epithelial origin, which included 11 lung cancers, two breast cancers, six stomach cancers, two hepatomas, six ovarian cancers, two pancreatic cancers, two colon cancers, one urinary bladder cancer and one vulvar epidermoid cancer (Table I). MS-2761 showed reactivity with one (HUVEC) of two cell lines of endothelial origin (Figure 1). Two fibroblast and three haematopoietic tumour cell lines examined did not express the antigen defined by MS-2761. The results of immunoperoxidase staining and slot-blot analysis completely matched each other with regard to the reactivity of MS-2761.

In immunoperoxidase staining, almost 100% of cells were stained strongly with MS-2761 in the primary culture of non-neoplastic mesothelial cells, but only 10–20% of the cells were stained in mesothelioma cell line NCC-MS-1 and hepatoma cell line NCC-Li21.

**Figure 1** Slot-blot analysis. A representative result of slot-blot analysis is shown. Five micrograms of protein of lysate of non-neoplastic mesothelial cells (A1) and 10 μg of protein of cell lysate of mesothelioma cell line NCC-MS-1 (A2), Cos-7 (A3), K234 (A4), K562 (A5), HL60 (A6), PC3 (A7), PC7 (A8), PC9 (A9), PC1 (A10), PC10 (A11), PC13 (B1), NCC-Lu99 (B2), NCC-Lu65 (B3), RMG1 (B4), RMG2 (B5), MCAS (B6), HUVEC (B7), TIG3 (B8), A431 (B9), PA1 (B10), PaCA-2 (B11), HCT15 (C1) and NCC-Li7 (C2) was blotted onto a nitrocellulose membrane using a filtration manifold. In slots C3–C11, no samples were loaded. Blots were detected by monoclonal antibody MS-2761 and an ECL Western blotting detection system (BioRad). Non-neoplastic mesothelial cells (A1), non-neoplastic endothelial cells (HUVEC) (B7) and mesothelioma cell line NCC-MS-1 (A2) are reactive with monoclonal antibody MS-2761, but other cell lines are not. No signal is evident in the negative control (data not shown).

**Table I** Reactivity of monoclonal antibody MS-2761 with cultured non-neoplastic and neoplastic cells *in vitro*

| *Tissue of origin* | *Positive (examined)* | *Percentage* |
|---|---|---|
| Non-neoplastic | | |
| Mesothelium | 6/6 | 100 |
| Endothelium | 1/2 | 50 |
| Fibroblast | 0/2 | 0 |
| Neoplastic | | |
| Mesothelioma | 1/1 | 100 |
| Lung cancer | 0/11 | 0 |
| Ovary cancer | 0/6 | 0 |
| Stomach cancer | 0/6 | 0 |
| Pancreatic cancer | 0/2 | 0 |
| Colon cancer | 0/2 | 0 |
| Breast cancer | 0/2 | 0 |
| Bladder cancer | 0/1 | 0 |
| Liver cancer | 1/3 | 33 |
| Vulva cancer | 0/1 | 0 |
| Hematopoietic | 0/3 | 0 |

Summary of immunoperoxidase staining and slot-blot analysis. Experiments were repeated at least twice to confirm the consistency of the results. The results of immunoperoxidase staining and slot-blot analysis of these cultured cells coincided with each other.

*Immunohistochemical reactivity of MS-2761 (Figures 2 and 3 and Table II)*

In 12 cases of pleural mesothelioma, histopathologically diagnosed at the National Cancer Center Hospital, Tokyo, acetone-fixed and paraffin-embedded tissues (AMeX; Sato *et al*., 1986) were available and and selected for this study. Among the 12 cases, five were classified as benign fibrous mesothelioma of the pleura. Histologically, benign fibrous mesothelioma is characterised by abundant dense collagenous materials. Immunohistochemically, MS-2761 reacted with the surfaces of mesothelioma cells, forming a network in the dense fibrous stroma (Figure 2a). Among the seven cases of malignant mesothelioma, two were classified as monophasic epithelioid or fibrous mesothelioma and five as biphasic mesothelioma. In epithelioid mesothelioma or the epithelioid component of biphasic mesothelioma, the MS-2761 antigen was expressed on the inner surface of the gland-like spaces formed by the tumour cells (Figure 2b) or in the cytoplasm of the tumour cells (Figure 2c). When compared with epithelioid mesothelioma, the staining intensity of fibrous mesothelioma or the sarcomatous component of biphasic mesothelioma was relatively faint and the cytoplasm of the tumour cells was stained (Figure 2d).

As shown in Table II, all the epithelial tumour cells except for one small-cell lung cancer (77/78) were immunohistochemically negative for the MS-2761 antigen. These tumours included lung adenocarcinoma, breast cancer, ovarian cancer and stomach cancer, all of which are known to metastasise frequently or spread to the pleura or peritoneum (Chernow and Sahn, 1977) and often create problems of differential diagnosis from mesothelioma. One small-cell lung cancer positive for MS-2761 was from a patient who was given extensive chemotherapy prior to surgical resection of the tumour. This cancer showed intercellular membranous staining with MS-2761 (Figure 2e). This staining pattern was not observed in epithelioid mesothelioma. In one case of lung adenocarcinoma, a small number (<5%) of tumour cells at the edge (or the proliferation tip) showed membranous staining (Figure 2f).

**Figure 2** Immunohistochemical detection of MS-2761 antigen in human tumours. Immunoperoxidase staining (ABC method; Hsu *et al*., 1981) was performed on AMeX tissue sections (Sato *et al*., 1986). (**a**) Benign fibrous mesothelioma. Cell membrane of tumour cells is stained with monoclonal antibody MS2761. (**b**) Malignant epithelioid mesothelioma. The inner surface of the gland-like spaces formed by tumour cells is stained. (**c**) Malignant epithelioid mesothelioma. Tumour cells show cytoplasmic granular staining. (**d**) Malignant fibrous mesothelioma. Cytoplasmic staining is detected in the tumour cells. (**e**) Small-cell lung cancer. In one out of six small-cell lung cancers (16%) intercellular membranous staining is observed, in contrast to the staining pattern of mesothelioma. (**f**) Lung adenocarcinoma. Intercellular staining is evident only in one cell nest at the edge (or proliferation tip) of this tumour (arrow). Magnification: **a** and **d** × 90; **b**, **c** and **f** × 180; **e** × 360.

Although tumour cells were not stained with MS-2761, strong staining was observed in the fibrous stroma of most epithelial tumours (Figure 3a).

In non-neoplastic tissues obtained at autopsy, mesothelium (Figure 3b), vascular endothelium (Figure 3c), epithelium of Bowman's capsule (Figure 3d), Kupffer cells of liver sinusoids (Figure 3e), dendric fibres of lymph follicles and adrenal cortex cells (Figure 3f) were stained with MS-2761. When hybridoma supernatant was replaced by normal mouse IgG, no staining was observed in these tissues (data not shown).

*Immunoprecipitation (Figure 4)*

In order to determine the molecular weight and *N*-glycosylation status of the antigen in mesothelium defined by monoclonal antibody MS-2761, immunoprecipitation and subsequent *N*-glycosidase treatment were performed. $^{35}S$-Labelled cell lysate of cultured non-neoplastic mesothelial cells was immunoprecipitated by MS-2761 antibody and subjected to SDS-PAGE and autoradiography. Under reducing conditions, a major and rather broad band at 106–112 kDa and a minor and sharp band at 97 kDa were detected (Figure 4, lane A). After *N*-glycosidase treatment, which cleaves between the innermost *N*-acetylglicosamine (GlcNAc) and asparagine residue of glycoprotein (Tarentino *et al.*, 1990), a single band at 80–82 kDa was detected (Figure 4, lane B). This suggested that the antigen molecule defined by MS-2761 has two different forms of *N*-glycosylation.

*Molecular cloning of the gene that encodes MS-2761 glycoprotein*

The potential value for differential diagnosis of mesothelioma and its unique tissue distribution encouraged us to carry out molecular cloning of the gene encoding the cell-surface glycoprotein defined by this monoclonal antibody. Because the antigenicity was labile to heat denaturation, SDS or a high concentration of urea (data not shown), monoclonal antibody MS-2761 was considered to recognise the confor-

**Figure 3** Immunohistochemical detection of MS-2761 antigen in human tissues. Immunoperoxidase staining (ABC method; Hsu *et al.*, 19981) was performed on AMeX tissue sections (Sato *et al.*, 1986). (**a**) Lung adenocarcinoma. Expression of MS2761 antigen is observed in the fibrous cancer stroma but not in any of the cancer cells. (**b**) Visceral pleura of the lung. Mesothelial cells covering the serous surface of the lung and subpleural vascular endothelial cells are stained. (**c**) Lung. Vascular endothelial cells are stained, but no staining is evident in alveolar cells. (**d**) Kidney. Epithelium of Bowman's capsule is stained. (**e**) Liver. The cytoplasm of Kupffer cells in liver sinusoids is stained, but liver parenchymal cells are unstained. (**f**) Adrenal cortex. Cell membrane of adrenal cortex cells is stained. Magnification: **a** and **c** × 90; **b** and **d** × 360; **e** and **f** × 180.

**Table II** Immunohistochemical reactivity of monoclonal antibody MS-2761 with human tumour tissues

| *Organ of origin* / *Histological subtype* | *Positive/ examined* | *Percentage* |
|---|---|---|
| Mesothelioma | | |
| Benign, fibrous | 5/5 | 100 |
| Malignant, epithelioid | 1/1 | 100 |
| Malignant, fibrous | 1/1 | 100 |
| Malignant, biphasic | 5/5 | 100 |
| Lung cancer | | |
| Adenocarcinoma | 0[a]/18 | 0 |
| Squamous cell carcinoma | 0/5 | 0 |
| Large-cell carcinoma | 0/4 | 0 |
| Small-cell carcinoma | 1/6 | 16 |
| Metastatic lung tumour | | |
| Colorectal carcinoma | 0/2 | 0 |
| Breast cancer | | |
| Invasive ductal carcinoma | 0/22 | 0 |
| Medullary carcinoma | 0/1 | 0 |
| Ovarian tumour | | |
| Mucinous cystadenoma | 0/1 | 0 |
| Serous papillary adenocarcinoma | 0/4 | 0 |
| Mucinous cystic adenocarcinoma | 0/2 | 0 |
| Clear cell adenocarcinoma | 0/1 | 0 |
| Malignant clear cell adenofibroma | 0/1 | 0 |
| Immature teratoma | 0/1 | 0 |
| Stomach cancer | | |
| Adenocarcinoma | 0/10 | 0 |

[a]In one case of lung adenocarcinoma, a small number (<5%) of tumour cells at the edge (or the proliferation tip) showed membrane staining (Figure 2f).

**Figure 4** Immunoprecipitation of MS-2761 antigen and subsequent *N*-glycosidase treatment. $^{35}$S-Labelled cell lysate of non-neoplastic mesothelial cells was immunoprecipitated by monoclonal antibody MS-2761 (lanes A and B) or normal mouse IgG (lanes C and D). Molecular weights (in kDa) were determined by sodium dodecyl sulphate polyacrylamide gel electrophoresis (SDS–PAGE) under reducing conditions and autoradiography. Without N-glycosidase treatment, a major broad band at 106–112 kDa and a minor one at 97 kDa are detected (lane A). After treatment of the immunoprecipitates with *N*-glycosidase, a single band 80–82 kDa in size is evident (lane B). When monoclonal antibody MS-2761 is replaced by normal mouse IgG, no signal is detected (lanes C and D).

mational structure of the antigen molecule. Therefore, we decided to construct a cDNA library to express the antigen in Cos-7 monkey kidney cells and select cDNA clones by panning, as described by Seed and Aruffo (1987). After the third panning, restriction digestion analysis revealed that cDNA clones with insert sizes of 3.2 kb and 2.8 kb had become prominent. Among 15 clones picked randomly, five clones had identical inserts 3.2 kb in size and four had identical inserts of 2.8 kb. By transfecting into Cos-7 cells and subsequent immunoperoxidase staining, two representative clones, pMS2761-32 (3.2 kb) and pMS2761-28 (2.8 kb), were found to contain the cDNA of the molecule possessing the MS-2761 antigenic determinant. Cos-7 cells transfected with the pCDNA1/Amp vector alone were not reactive with MS-2761.

### *DNA sequencing and database search*

As shown in Figure 5, DNA sequencing and restriction mapping revealed that the two cDNA clones shared an identical 5′ end sequence and an identical open reading frame but different polyadenylation sites. A database search revealed that the nucleotide sequence was identical to the cDNA of vascular cell adhesion molecule 1 (VCAM1), which was originally cloned from an expression library of cytokine-activated endothelial cells (Osborn *et al.*, 1989). Both pMS2761-32 and pMS2761-28 had four additional nucleotides (GCAT) at the 5′ end of the registered VCAM1 cDNA sequence, but lacked seven nucleotides from the transcriptional start site determined by primer extension analysis (Cybulsky *et al.*, 1991*b*).

### *Northern blotting (Figure 6)*

mRNA expression of non-neoplastic mesothelial cells from two individuals, mesothelioma cell NCC-MS-1, ovarian cancer cell line TYKnu, lung cancer cell line NCC-Lu99 and liver cancer cell line NCC-Li21 was electrophoresed and hybridised with a radiolabelled 1.5 kb 5′ fragment of the pMS2761-28 insert (Figure 5). Non-neoplastic mesothelial cells expressed a predominant 3.2 kb transcript (Figure 6, lanes A and B). The VCAM1 expression of mesothelioma cell line NCC-MS-1 (lane C) and liver cancer cell line NCC-Li21 (lane F) was much lower than that of non-neoplastic mesothelial cells. TYKnu (lane D) and NCC-Lu99 (lane E) did not express VCAM1 mRNA. In addition to a 3.2 kb transcript, 7.5 and 5.0 kb mRNAs, not described before, were detected in non-neoplastic mesothelial cells (Figure 6, lanes A and B). These results were consistent with the reactivity of these cells with monoclonal antibody MS-2761.

**Figure 5** Structure of human VCAM1 cDNA, pMS2761-32 and pMS2761-28. The schematic structure of two cDNA clones, pM2761-32 and pMS2761-28, obtained in this study is presented, in comparison with the registered VCAM1 cDNA sequence (top). pM2761-32 and pMS2761-28 have identical 5′ side sequences but different polyadenylation sites. The 3′ end sequence preceding the polyadenylation site of pMS2761-28 is shown. The consensus polyadenylation signal AATAAA is underlined. A 1504 bp 5′ fragment of the pMS2761-28 insert (bottom) was used as a probe for Northern blotting.

*RT-PCR (Figure 7)*

In endothelial cells, alternative splicing results in two different mRNAs encoding VCAM1 with six or seven Ig-like domains (Cybulsky *et al.*, 1991*a*; Hession *et al.*, 1991). In order to determine if the VCAM1 in non-vascular cells has six or seven Ig-like domains, the region covering domains 3 and 4 was amplified (Figure 7), where alternative splicing takes place in cytokine-activated endothelial cells. Non-neoplastic mesothelial cells (lane A), mesothelioma cells NCC-MS-1 (lane B) and NCC-Li21 hepatoma cells (lane E) expressed both a predominent 631 bp PCR fragment and an additional 355 bp PCR fragment, suggesting coexpression of two types of transcripts with six and seven Ig-like domains. The expression level was lower in NCC-MS-1 and NCC-Li21 than in non-neoplastic mesothelial cells. Expression of VCAM1 mRNA in two other epithelial tumour cell lines, TYKnu (lane C) and NCC-Lu99 (lane D), could not be detected, even using such a sensitive method. These results of RT-PCR are consistent with those of Northern blotting and the reactivity of these cells with monoclonal antibody MS-2761 described above.

### Discussion

Vascular cell adhesion molecule 1 [VCAM1, also referred to as INCAM110 (Rice and Bevilacqua, 1989)] is a member of the immunoglobulin (Ig) superfamily. VCAM1 cDNA was originally separated from cytokine-activated endothelial cells by Osborn *et al.* (1989). VCAM1 expression on resting endothelial cells is minimal, but it can be induced by various stimuli, including interleukin 1$\beta$ (IL-1$\beta$) and tumour necrosis factor alpha (TNF-$\alpha$) (Osborn *et al.*, 1989). Through its interaction with integrin $\alpha_4\beta_1$ (Elices *et al.*, 1990), VCAM1 acts as an adhesion molecule of endothelial cells to lymphocytes, monocytes, eosinophils and basophils, and is thought to initiate the extravasation of these inflammatory cells (Briscoe *et al.*, 1992). The expression of VCAM1 in inflammatory sites suggests its involvement in the pathogenesis of inflammatory diseases (Rice *et al.*, 1991).

Non-vascular cells expressing VCAM1 are reported to include follicular dendritic cells of lymph nodes, bone marrow stroma cells and synovial cells (Freedman *et al.*, 1990; Rice *et al.*, 1991; Simmons *et al.*, 1992). Taking into consideration the morphological and functional similarity between endothelium and mesothelium, it is not surprising that endothelium and mesothelium share such a cell-surface molecule. The *in vivo* expression of VCAM1 in mesothelium was described by Rice *et al.* (1991), but it has not been examined in further detail, except for a few sporadic *in vitro* studies (Jonjic *et al.*, 1992; Cannistra *et al.*, 1993). In the present search for a new marker for discriminating mesothelium from epithelium, we produced a monoclonal antibody and identified VCAM1 as its antigen molecule by expression cDNA cloning. We also demonstrated the selective reactivity of monoclonal antibody MS-2761 with non-neoplastic mesothelium and mesothelioma both *in vitro* and *in vivo*. Non-neoplastic mesothelial cells obtained from the peritoneal omentum of six individuals uniformly expressed MS-2761 antigen. All the cases of benign and malignant or fibrous and epithelial mesothelioma examined in this study reacted with this monoclonal antibody, but 77 out of 78 epithelial tumour tissues and 33 out of 34 epithelial tumour cell lines were not reactive. Furthermore, the specific expression of VCAM1 glycoprotein in non-neoplastic mesothelium and mesothelioma was confirmed at the RNA level by Northern blotting and RT-PCR. The high specificity of MS-2761 for mesothelium suggests the potential usefulness of this anti-VCAM1 antibody for differential diagnosis of mesothelioma.

Cultured non-neoplastic mesothelial cells constitutively express VCAM1 even without cytokine stimuli. The physiological role of the VCAM1 molecule on the cell surface of the mesothelium has not yet been explored and remains to be investigated.

In Northern blotting, a prominent 3.2 kb transcript of VCAM1 was detected in non-neoplastic mesothelial cells. In addition, two minor transcripts, 5.0 and 7.5 kb in size, were also detected. These longer transcripts have not been described in the literature and are now under investigation.

RT-PCR revealed that non-neoplastic mesothelial cells expressed VCAM1 with both of six and seven Ig-like domains, similar to cytokine-activated endothelial cells (Cybulsky *et al.*, 1991*a*; Hession *et al.*, 1991). In this study, two near-full length cDNAs of VCAM1 were separated by expression cDNA cloning. These two cDNA clones shared identical 5' end sequences but had different polyadenylation sites. It is likely that the shorter cDNA clone was the result of DNA recombination during the cloning procedures including repeated transformation into *E. coli* and DNA transfection into Cos-7 monkey cells. However, the shorter cDNA clone had a sequence identical to the longer one and a polyadenylation site proceded by the consensus poly(A) signal sequence, AATAAA. This implies the presence of a minor differently polyadenylated population of VCAM1 mRNA which has not been detected before.

The expression level of VCAM1 in mesothelioma cell line NCC-MS-1 was much lower than its its non-neoplastic counterparts. In fact, cytogenetic analysis has revealed that the most frequent change seen in malignant mesothelioma is deletion of chromosome 1p (Taguchi *et al.*, 1993), which bears the VCAM1 gene (Cybulsky *et al.*, 1991*b*). Deletion of

**Figure 6** Northern blotting. Expression of VCAM1 mRNA was examined in non-neoplastic mesothelial cells from two individuals (lanes A and B), mesothelioma cell line NCC-MS-1 (lane C), lung cancer cell line NCC-Lu99 (lane D), ovarian cancer cell line TYKnu (lane E) and hepatoma cell line NCC-Li21 (lane F) by Northern blotting. Non-neoplastic mesothelial cells express a prominent transcript 3.2 kb in size and two minor transcripts of 5.0 and 7.5 kb. NCC-MS-1 and NCC-Li21 also express the 3.2 kb transcript. Two epithelial tumour cell lines, NCC-Lu99 and TYKnu, do not express VCAM1 mRNA. The quality and quantity of electrophoresed mRNAs were determined by rehybridisation of the same blot with a human glyceraldehyde-3-phosphate dehydrogenase (G3PDH) cDNA probe (bottom).

**Figure 7** RT-PCR for the detection of seven and six Ig-like domain forms of VCAM1 mRNA. First-strand cDNA of non-neoplastic mesothelial cells (lanes B and G), mesothelioma cell line NCC-MS-1 (lanes C and H), lung cancer cell line NCC-Lu99 (lanes D and I), ovarian cancer cell line TYKnu (lanes E and J) and hepatoma cell line NCC-Li21 (lanes F and L) was amplified by PCR and analysed by agarose gel electrophoresis and ethidium bromide staining. VCAM1 cDNA fragments of 631 bp (seven Ig-like domains) and 335 bp (six Ig-like domains) were amplified from the first-strand cDNA of non-neoplastic mesothelial cells, NCC-MS-1 and NCC-Li21 but not from NCC-Lu99 or TYKnu (lanes B through F). Lanes G-K show amplification of a 721 bp fragment of $\beta$-actin cDNA in these cells. Lanes A and L, molecular weight marker (100 bp ladder, Pharmacia).

a specific site on 1p may be responsible for partial down-regulation of VCAM1 expression during the course of oncogenesis. In our preliminary examination, however, no apparent abnormality of the VCAM1 gene was detected in NCC-MS-1 by Southern blotting (data not shown). Further detailed studies on the mechanism of down-regulation of VCAM1 gene expression appear to be necessary.

The tissue distribution of MS-2761 antigen was quite similar to that of VCAM1 reported in the literature. However, through immunohistochemical analysis, we found that the antigen defined by MS-2761 was strongly expressed in cancer stroma. Histologically, cancer often shows massive infiltration of inflammatory cells such as lymphocytes, plasma cells and basophils. VCAM1 may act as an inducer of these inflammatory cells into the cancer stroma. Cancer cells and stroma interact with each other either directly or through cytokine production. The expression of VCAM1 in cancer stroma, described in this paper for the first time, may play a role in the growth and invasion of cancer. We are currently investigating the functional and molecular characteristics of VCAM1 in cancer stroma.

Unlike diffuse malignant mesothelioma, the histogenesis of benign localised fibrous mesothelioma (or localised fibrous tumour of the serous cavities) still remains controversial. There is no conclusive evidence to indicate whether this tumour is of mesothelial cell or of submesothelial connective tissue origin. As MS2761 is reactive with cancer stromal fibroblasts, it is likely that it reacts with some activated mesenchymal cells. Thus, the possibility of benign fibrous mesothelioma being of mesenchymal origin is not ruled out solely because of its reactivity with MS-2761. Furthermore, in fibrous mesothelioma or the sarcomatous component of biphasic mesothelioma, the cytoplasmic staining of tumour cells was indistinguishable from that of activated fibroblasts in cancer stroma.

In conclusion, we have developed a new monoclonal antibody for differential diagnosis of mesothelioma. Through molecular cloning, we have identified the antigenic molecule as VCAM1 in mesothelium. Detailed immunohistochemistry revealed that this antigen is expressed in mesothelium and mesotheliomas but not epithelium or most tumours of epithelial origin. Currently, surgical pathologists use many immunohistochemical markers in routine diagnosis. Many immunoglobulin superfamily members such as CEA and NCAM (Patel *et al.*, 1989) are among those used most commonly. This is probably due to the relatively restricted tissue distribution of this family. Although a larger series of tumours with different histological patterns needs to be studied to confirm the specificity, this monoclonal antibody seems to have a potential value for discrimination of mesothelium and epithelium, and further studies of VCAM1 should provide insight into the functional properties of mesothelium.

**Acknowledgements**

We thank Drs H Tsuda, Y Kanai, T Oda and T Shibata for useful advice and Ms Yamauchi for tissue preparation. We are also grateful to the staff of the Photo Center, National Cancer Center, Tokyo, for excellent photographic assistance. This study was supported in part by a Grant-in-Aid for Cancer Research from the Ministry of Health and Welfare of Japan.

**References**

ADEN DP, FOGEL A, PLOTKIN S, DAMJANOV I AND KNOWLES BB. (1979). Controlled synthesis of HBsAg in a differentiated human liver carcinoma-derived cell line. *Nature*, **282**, 615–616.

BRISCOE DM, COTRAN RS AND POBER JS. (1992). Effects of tumor necrosis factor, lipopolysaccharide, and IL-4 on the expression of vascular cell adhesion molecule-1 in vivo. Correlation with CD3$^+$ T cell infiltration. *J. Immunol.*, **149**, 2954–2960.

BUBENIK J, BARESOVA M, VIKLICKY V, JAKOUBKOVA J, SAINEROVA H AND DONNER J. (1973). Established cell line of urinary bladder carcinoma (T24) containing tumor-specific antigen. *Int J Cancer*, **11**, 765–773.

CANNISTRA SA, KANSAS GS, NILOFF J, DEFRANZO B, KIM Y AND OTTENSMEIER C. (1993). Binding of ovarian cancer cells to peritoneal mesothelium *in vitro* is partly mediated by CD44H. *Cancer Res.*, **53**, 3830–3838.

CARNEY DN, GAZDAR AF, BEPLER G, GUCCION JG, MARANGOS PJ, MOODY TW, ZWEIG MH AND MINNA JD. (1985). Establishment and identification of small cell lung cancer cell lines having classic and variant features. *Cancer Res.*, **45**, 2913–2923.

CHANG K, PAI LH, PASS H, POGREBNIAK HW, TSAO MS, PASTAN I, AND WILLINGHAM MC. (1992). Monoclonal antibody K1 reacts with epithelial mesothelioma but not with lung adenocarcinoma. *Am. J. Surg. Pathol.*, **16**, 259–268.

CHERNOW B AND SAHN SA. (1977). Carcinomatous involvement of pleura. An analysis of 96 patients. *Am. J. Med.*, **63**, 695–702.

CRAIGHEAD JE AND MOSSMAN BT. (1982). The pathogenesis of asbestos related disease. *N. Engl. J. Med.*, **306**, 1446–1455.

CYBULSKY MI, FRIES JWU, WILLIAMS AJ, SULTAN P, DAVIS VM, GIMBORNE Jr MA AND COLLINS T. (1991*a*). Alternative splicing of human VCAM-1 in activated vascular endothelium. *Am. J. Pathol.*, **138**, 815–820.

CYBULSKY MI, FRIES JWU, WILLIAMS AJ, SULTAN P, EDDY R, BYERS M, SHOWS T, GIMBORNE Jr MA AND COLLINS T. (1991*b*). Gene structure, chromosomal location, and basis for alternative mRNA splicing of the human *VCAM1* gene. *Proc. Natl Acad. Sci. USA*, **88**, 7859–7863.

DONNA A, BETTA PG AND JONES JSP. (1988). Verification of the histologic diagnosis of malignant mesothelioma in relation to the binding of an antimesothelial cell antibody. *Cancer*, **63**, 1331–1336.

ELICES MJ, OSBORN L, TAKADA Y, CROUSE C, LUHOWSKYJ S, HEMLER ME AND LOBB RR. (1990). VCAM-1 on activated endothelium interacts with the leukocyte integrin in VLA-4 at a site distinct from the VLA-4 fibronectin binding site. *Cell*, **60**, 577–584.

FREEDMAN AS, MUNRO JM, RICE GE, BEVILACQUA MP, MORIMOTO C, MACINTYRE BW, RHYNHART K, POBER JS AND NADLER LM. (1990). Adhesion of human B cells to germinal centers in vitro involves VLA-4 and INCAM-110. *Science*, **249**, 1030–1033.

GIARD DJ, AARONSON SA, TODARO GJ ARNSTEIN P, KERSEY JH, DOSIK H AND PARKS WP. (1973). *In vitro* cultivation of human tumors: establishment of cell lines derived from a series of solid tumors. *J. Natl Cancer Inst.*, **51**, 1417–1423.

GLUZMAN Y. (1981). SV40-transformed simian cells support the replication of early SV40 mutants. *Cell*, **23**, 175–182.

HAYFLICK L AND MOORHEAD PS. (1961). The serial cultivation of human diploid cell strains. *Exp. Cell Res.*, **25**, 585–621.

HESSION C, TIZARD R, VASSALLO C, SCHIFFER SB, GOFF D, MOY P, CHI-ROSSO G, LUHOWSKYJ S, LOBB RR AND OSBORN L. (1991). Cloning of an alternate form of vascular cell adhesion molecule-1 (VCAM1). *J. Biol. Chem.*, **266**, 6682–6685.

HIRANO T, HIROHASHI S, KUNII T, NOGUCHI M, SHIMOSATO Y AND HAYATA Y. (1989). Quantitative distribution of cluster 1 small cell lung cancer antigen in cancerous and non-cancerous tissues, cultured cells and sera. *Jpn J. Cancer Res.*, **80**, 348–355.

HIRT B. (1967). Selective extraction of polyoma DNA from infected mouse cell culture. *J. Mol. Biol.*, **26**, 365–369.

HSU SM, RAINE L AND FANGER H. (1981). Use of avidin-biotin-peroxidase complex (ABC) in immunoperoxidase techniques. A comparison between ABC and unlabeled (PAP) procedures. *J. Histochem. Cytochem.*, **29**, 577–580.

HSU SM, HSU PL, ZHAO X, KAO-SHAN CS AND WHANG-PENG J. (1988). Establishment of human mesothelioma cell line (MS-1, -2) and production of a monoclonal antibody (Anti-MS) with diagnostic and therapeutic potential. *Cancer Res.*, **48**, 5228–5236.

JONJIC N, PERI G, BERNASCONI S, SCIACCA FL, COLOTTA F, PELICCI P, LANFRANCONE L AND MANTOVANI A. (1992). Expression of adhesion molecules and chemotactic cytokines in cultured human mesothelial cells. *J. Exp. Med.*, **176**, 1165–1174.

KERN PA, KNEDLER A AND ECKEL RH. (1983). Isolation and culture of microvascular endothelium from human adipose tissue. *J. Clin. Invest.*, **71**, 1822–1829.

LAEMMLI UK. (1970). Cleavage of structural proteins during the assembly of the head of bacteriophage T4. *Nature*, **227**, 680–685.

O'HARA CJ, CORSON JM, PINKUS GS AND STAHEL RA. (1990). ME1 A monoclonal antibody that distinguishes epithelial-type malignant mesothelioma from pulmonary adenocarcinoma and extrapulmonary malignancies. *Am. J. Pathol.*, **136**, 421–428.

OSBORN L, HESSION C, TIZARD R, VASSALLO C, LUHOWSKYJ S, CHI-ROSSO G AND LOBB R. (1989). Direct expression cloning of vascular cell adhesion molecule 1, a cytokine-induced endothelial protein that binds to lymphocytes. *Cell*, **59**, 1203–1211.

PATEL K, MOORE SE, DICKSON G, ROSSELL RJ, BEVERLY PC, KEMSHEAD JT AND WALSH FS. (1989). Neural cell adhesion molecule (NCAM) is the antigen recognized by monoclonal antibodies of similar specificity in small-cell lung carcinoma and neuroblastoma. *Int. J. Cancer*, **44**, 573–578.

RICE GE AND BEVILACQUA MP. (1989). An inducible endothelial cell surface glycoprotein mediates melanoma adhesion. *Science*, **246**, 1303–1306.

RICE GE, MUNRO JM, CORLESS C AND BEVILACQUA MP. (1991). Vascular and nonvascular expression of INCAM-110. A target for mononuclear leukocyte adhesion in normal and inflamed human tissues. *Am. J. Pathol.*, **138**, 385–393.

SAMBROOK J, FRITSCH EF AND MANIATIS T. (1989). *Molecular Cloning. A Laboratory Manual*, 2nd edn. Cold Spring Harbor Laboratory Press: Plainview, NY.

SANGER F, NICKLE S AND COULSON AR. (1977). DNA sequencing with chain-terminating inhibitors. *Proc. Natl. Acad. Sci. USA*, **74**, 5463–5467.

SATO Y, MUKAI K, WATANABE S, GOTOH M AND SHIMOSATO Y. (1986). The AMeX method. A simplified technique of tissue processing and paraffin embedding with improved preservation of antigens for immunostaining. *Am. J. Pathol.*, **125**, 431–435.

SEED B AND ARUFFO A. (1987). Molecular cloning of the CD2 antigen, the T-cell erythrocyte receptor, by a rapid immunoselection procedure. *Proc. Natl Acad. Sci. USA*, **84**, 3365–3369.

SHEIBANI K, ESTEBAN JM, BAILEY A, BATTIFORA H AND WEISS LM. (1992). Immunopathologic and molecular studies as an aid to the diagnosis of malignant mesothelioma. *Hum. Pathol.*, **23**, 107–116.

SIMMONS PJ, MASINOVSKY B, LOGENECKER BM, BERENSON R, TOROK-STORB B AND GALLATIN WM. (1992). Vascular cell adhesion molecule-1 expressed by bone marrow stromal cells mediates the binding of hematopoietic progenitor cells. *Blood*, **80**, 388–395.

SOULE HD, VAZQUEZ J, LONG A, ALBERT S AND BRENNAN M. (1973). A human cell line from a pleural effusion derived from a breast carcinoma. *J. Natl Cancer Inst.*, **51**, 1409–1416.

STAHEL RA, O'HARA CJ, WAIBEL R AND MARTIN A. (1988). Monoclonal antibodies against mesothelial membrane antigen discriminate between malignant mesothelioma and lung adenocarcinoma. *Int. J. Cancer*, **41**, 218–223.

TAGUCHI T, JHANWAR SC, SIEGFRIED JM, KELLER SM AND TESTA JR. (1993). Recurrent deletions of specific chromosomal sites in 1p, 3p, 6q and 9p in human malignant mesothelioma. *Cancer Res.*, **53**, 4349–4355.

TAKAHASHI K, SAWASAKI Y, GOTO T, HATA J AND MUKAI K. (1989). Cobblestone monolayer cells from human omental adipose tissue are possibly mesothelial not endothelial. *In vitro Cell Dev. Biol.*, **25**, 109–111.

TAKAHASHI K, SAWASAKI Y, HATA J, MUKAI K AND TOTO T. (1990). Spontaneous transformation and immortalization of human endothelial cells. *In vitro Cell Dev. Biol.*, **25**, 265–274.

TARENTINO AL, QUINONES G, TRUMBLE A, CHANGCHIEN L-M, DUCEMAN B, MALEY F AND PLUMMER TH. (1990). Molecular cloning and amino acid sequence of peptide-$N^4$-(*N*-acetyl-β-D-glucosaminyl) asparagine amidase from *Flavobacterium meningosepticum*. *J. Biol. Chem.*, **265**, 6961–6966.

WARHOL MJ, HICKEY WF AND CORSON JM. (1982). Malignant mesothelioma: ultrastructural distinction from adenocarcinoma. *Am. J. Surg. Pathol.*, **6**, 307–314.

YAMADA T, HIROHASHI S, SHIMOSATO Y, KODAMA T, HAYASHI S, OGURA T, GAMOU S AND SHIMIZU N. (1985). Giant cell carcinomas of the lung producing colony-stimulating factor *in vitro* and *in vivo*. *Jpn J. Cancer Res.*, **76**, 967–976.

YAMADA T, HIROHASHI S, SHIMOSATO Y, WATANABE M, KAMIO S, KUNII T AND HAYATA Y. (1987). Monoclonal antibody raised by sera of athymic mice bearing human lung cancer xenografts. *Oncology*, **44**, 186–191.